# The Human IL-22 Receptor Is Regulated through the Action of the Novel E3 Ligase Subunit FBXW12, Which Functions as an Epithelial Growth Suppressor

**DOI:** 10.1155/2015/912713

**Published:** 2015-06-11

**Authors:** Joseph Franz, Jacob Jerome, Travis Lear, Qiaoke Gong, Nathaniel M. Weathington

**Affiliations:** ^1^George Washington University Medical School, Washington, DC 20037, USA; ^2^Department of Medicine, Acute Lung Injury Center of Excellence, University of Pittsburgh, Pittsburgh, PA 15213, USA

## Abstract

Interleukin- (IL-) 22 signaling is protective in animal models of pneumonia and bacteremia by *Klebsiella pneumoniae* and mediates tissue recovery from influenza and *Staph aureus* infection. We recently described processing of mouse lung epithelial IL-22 receptor (IL-22R) by ubiquitination on the intracellular C-terminal. To identify cellular factors that regulate human IL-22R, we screened receptor abundance while overexpressing constituents of the ubiquitin system and identify that IL-22R can be shuttled for degradation by multiple previously uncharacterized F-box protein E3 ligase subunits. We observe that in human cells IL-22R is destabilized by FBXW12. FBXW12 causes depletion of endogenous and plasmid-derived IL-22R in lung epithelia, binds the E3 ligase constituent Skp-1, and facilitates ubiquitination of IL-22R *in vitro*. FBXW12 knockdown with shRNA increases IL-22R abundance and STAT3 phosphorylation in response to IL-22 cytokine treatment. FBXW12 shRNA increases human epithelial cell growth and cell cycle progression with enhanced constitutive activity of map kinases JNK and ERK. These findings indicate that the heretofore-undescribed protein FBXW12 functions as an E3 ligase constituent to ubiquitinate and degrade IL-22R and that therapeutic FBXW12 inhibition may enhance IL-22 signaling and bolster mucosal host defense and infection containment.

## 1. Introduction

The activity of IL-22 cytokine signaling has been described as an important defense against Gram-negative pathogens in gastrointestinal [1] and pulmonary [2] systems through action on IL-22R, which is exclusively expressed on epithelia [3]. IL-22 signaling is protective in multiple models of infection and elicits antimicrobial peptides as well as protective cytokine responses in the setting of infection [4]. Ligation of IL-22R directs migration and proliferation of epithelia in these organs to fortify the barrier integrity via molecular events including phosphorylation of STAT3, MAP kinase activation, and activation of proliferative genes such as c-Myc and cyclin D1 [5–7]. In lung, IL-22 signaling is essential for the protective host response to* Klebsiella pneumoniae*, and IL-22 axis disruption worsens lung injury in the setting of staphylococcal and influenza respiratory infection in animal models [2, 8]. We recently demonstrated in mouse lung epithelia that ligation of the IL-22R causes phosphorylation of cytoskeletal effector cortactin, which is critical for cellular migration in response to IL-22 [9]. We also identified that IL-22R is rapidly depleted in response to IL-22 treatment and that murine IL-22R is ubiquitinated on its intracellular C-terminal domain to be degraded by the ubiquitin proteasome system [9].

The activity of the ubiquitin proteasome system is the major modality of homeostatic protein degradation within cells [10]. In this system, E1 enzymes process free ubiquitin (Ub) which is then loaded onto the E2 Ub conjugating enzymes. Substrate proteins are ubiquitinated on lysine amino acids through the action of E3 ligases, which usually bind specific substrate motifs and E2 molecules and facilitate Ub transfer onto substrate [11]. Polyubiquitinated substrate proteins are then recognized by the 26s proteasome, a barrel shaped protein complex which exhibits trypsin-, chymotrypsin-, and caspase-like proteolytic activities to degrade substrates.

There is a broad diversity of E3 ligases (hundreds) within the human genome including the Skp-1-cullin-F-box (SCF) family, in which the F-box proteins bind substrate and the Skp-1 and cullin constituents facilitate E2 approximation and Ub transfer. These F-box proteins are classified into three groups [12]: FBXL proteins contain a leucine rich domain, FBXW proteins harbor a WD-40 repeat, while FBXO family members possess neither of these domains. F-box proteins specifically bind (often to multiple) substrate proteins to be ubiquitinated and degraded. The activity and abundance of specific E3 ligases therefore can greatly sway the biology of cells based on the downstream changes in substrate abundance. This has been demonstrated for FBXW family members in neoplastic and inflammatory biology especially, with FBXW1 (also known as the beta transferrin repeat containing protein (*β*-trcp1)) and FBXW7 identified as likely oncogenes [13–15] whose deficiency or mutation is associated with multiple forms of cancer. Hence, components of the Ub system have been targeted for inflammatory and neoplastic diseases [16, 17]. Based on these observations, we sought to identify specific E3 ligases involved in the ubiquitination and degradation for the human proproliferative and proinflammatory cytokine receptor IL-22R.

## 2. Materials and Methods

### 2.1. Cell Cultures and Reagents

Immortalized human bronchial epithelial Beas-2B cells were purchased from the ATCC and maintained in HITES medium supplemented with 10% FBS and transfected by Nucleofection with Lonza products; HeLa cells were maintained in Eagle's medium with 10% FBS and transiently transfected with Turbofect (Thermo). Cells were maintained in a 37°C incubator with 5% CO_2_ as described previously. Anti-IL-22R antibody was from Millipore. Skp-1, phospho-STAT3, STAT3, and phospho-ERK antibodies were from Cell Signaling. V5 antibody, mammalian expression plasmid pcDNA3.1/HisV5-topo, and* Escherichia coli* Top10 competent cells were purchased from Life Technologies. Immobilized protein A/G beads were obtained from Pierce. Phospho-JNK and JNK antibodies are from Abcam. Cycloheximide, buffering chemicals, and phosphatase inhibitor mixtures were from Sigma. Gel extraction kits and QIAprep spin miniprep kits were from Qiagen.* In vitro* transcription and translation (TnT) kits were from Promega. DTT, ATP, ubiquitin activating enzyme, UbcH5, UbcH7, ubiquitin, and ubiquitin aldehyde were from Enzo Life Sciences (Farmingdale, NY). Skp-1, Rbx1, and Cul-1 were from Abnova (Taipei, Taiwan), and TnT Coupled Reticulocyte was from Promega (Madison, WI). Talon metal affinity resin was from Clontech (Mountain View, CA). All materials in the experiments are of the highest grade and commercially available.

### 2.2. Flow Cytometry

HeLa cells were transiently transfected as indicated and, after 36–48 hr, treated with 60 ng/mL recombinant human IL-22 or serum-free media alone for 8 h prior to staining with Vybrant Dyecycle Ruby (Life Technologies) per manufacturers' instructions. Cells were then analyzed on an Accuri cytometer and accompanying software.

### 2.3. Immunoprecipitation and Immunoblotting

Beas-2B and HeLa cells during exponential growth were treated with cytokine in serum-free media as indicated and were lysed with lysis buffer (0.3% Triton X-100 (v/v) in PBS and 1 : 1000 protease inhibitor mixture). Lysates were sonicated and centrifuged at 13,000 rpm for 10 min. For immunoprecipitations, cell lysates (containing 1 mg of protein) were incubated and rotated with 2 *μ*g of anti-V5 or anti-phosphoserine at 4°C for 4 hr and then incubated with 30 *μ*L of protein A/G-agarose beads for another 3 h and the beads were centrifuged and washed with lysis buffer three times. The washed beads were mixed with SDS-PAGE loading dye prior to SDS-PAGE and immunoblot analysis. Immunoblotting was performed as described previously [18].

### 2.4. Cell Growth Assay

HeLa cells were plated at 25% confluence and transfected with indicated plasmids (2 *μ*g plasmid DNA per well in 12-well Costar culture plates), and viable cell count was obtained with a BioRad TC20 automated cell counter at times indicated.

### 2.5. *In Vitro* Ubiquitin Conjugation Assay

The assay was performed at room temperature for 1 h in a volume of 25 *μ*L containing 50 mM Tris pH 7.6, 5 mM MgCl2, 0.6 mM DTT, 2 mM ATP, 300 *μ*M MG132, 50 nM ubiquitin activating enzyme, 0.5 *μ*M UbcH5, 0.5 *μ*M UbcH7, 2 *μ*M ubiquitin, and 1 *μ*M ubiquitin aldehyde, 20 nM Rbx1, 20 nM Cul-1, 20 nM Skp-1, and TnT Coupled Reticulocyte* in vitro* synthesized FBXW12-Myc and IL-22R-V5. TnT* in vitro* synthesized proteins were purified via Talon metal affinity resin and reaction products were processed for V5 immunoblotting.

### 2.6. Data and Statistical Analysis

All experiments shown are representative of at least 3 separate experiments performed on different days. Densitometry of representative immunoblots was performed using ImageJ software. We used InStat software to perform ANOVA analysis for Figures 3, 4, and 5.

## 3. Results

### 3.1. E3 Ligase Subunits of the F-Box W Family Cause IL-22R Degradation

**Figure 1:**
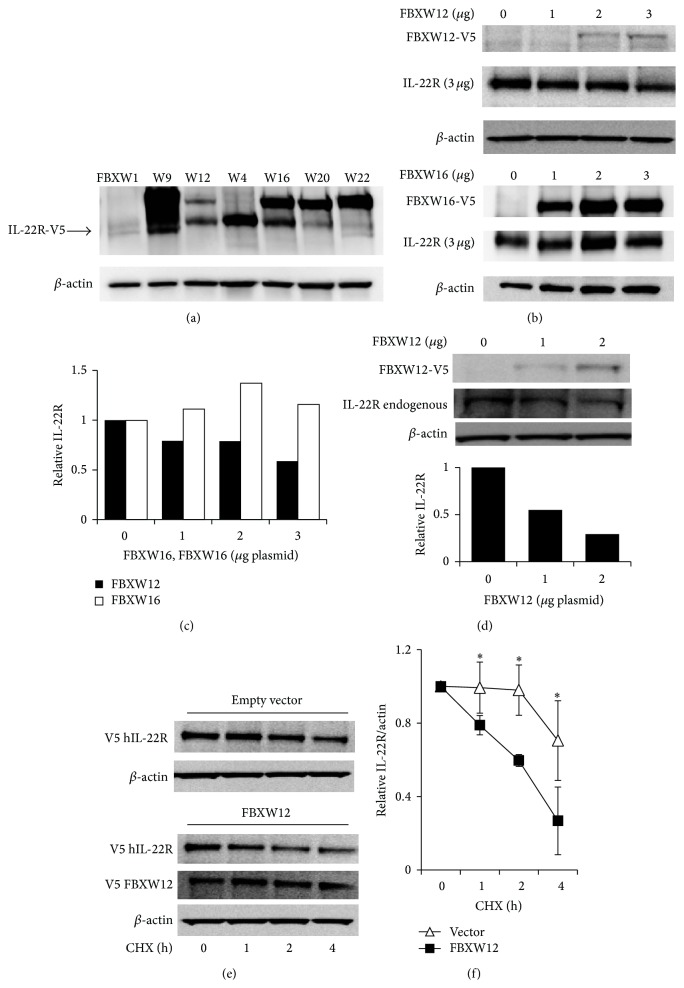
E3 ligase subunit FBXW12 decreases cellular levels of human IL-22Ra1. (a) Human IL-22Ra1-V5 plasmid (2 *μ*g) was transiently transfected into Beas-2B human airway epithelial-derived cells with V5-labeled E3 ligase subunits (2 *μ*g) FBXW1, FBXW9 (W9), and so forth and V5 immunoblot at 48 h is shown. (b) FBXW12-V5 and FBXW16-V5 were transfected at increasing doses with IL-22R and analyzed at 48 hr. Densitometric quantitation of (b) is shown in (c). (d) FBXW12-V5 was transfected into Beas-2B cells without IL-22R and IB for V5 (upper panel) and endogenous IL-22R are shown. (e) Beas-2B cells were transfected with IL-22R (2 *μ*g) and vector control or FBXW12 (2 *μ*g) and 48 h later treated with cycloheximide for time indicated; protein stability was assessed by IB and graphed (f). All data are representative of at least 3 separate experiments on 3 days (^*∗*^ *p* < 0.05 for FBXW12 versus vector by Student's* t*-test for *n* = 4).

We initially used Beas-2B cells to conduct a screen for IL-22R degradation in the setting of overexpression of multiple F-box proteins (Figure 1(a)). The well described E3 ligase constituent FBXW1 (*β*-trcp) degrades related cytokine receptors IL-10Ra1 and IFNAR and likewise causes a decrease in IL-22R abundance. The structurally homologous FBXW family members FBXW12, FBXW20, and FBXW22 each also decrease IL-22R-V5 signal when cooverexpressed with the receptor. IL-22R signal remains stable when FBXW16 and FBXW4 are overexpressed. Because FBXW20 and FBXW22 are murine proteins, we chose to more closely study the activity of the human F-box protein FBXW12 on human IL-22R. Human IL-22R was overexpressed with increasing doses of F-box protein-containing plasmids encoding FBXW12 or FBXW16 (Figure 1(b)), and only FBXW12 causes reduction in IL-22R levels (Figure 1(c)). When we overexpress FBXW12 alone and evaluate endogenous IL-22R, we likewise observe a dose dependent decrease in IL-22R abundance (Figure 1(d)). In the presence of the protein synthesis inhibitor cycloheximide, IL-22R levels fall over hours due to protein degradation with a half-life of approximately 6 hours. In the presence of overexpressed FBXW12, we observe an accelerated degradation of IL-22R with half-life closer to 2 hours (Figures 1(e) and 1(f)).

### 3.2. FBXW12 Functions as an E3 Ligase to Ubiquitinate IL-22R

**Figure 2:**
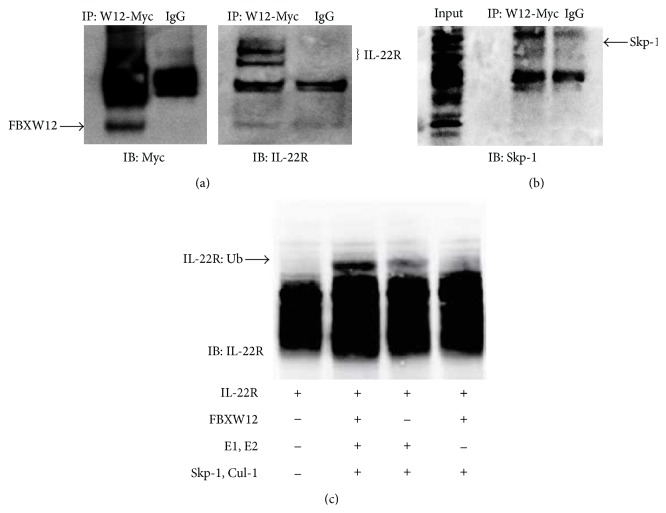
FBXW12 is functional as an E3 ligase subunit to ubiquitinate IL-22R. (a) FBXW12-Myc plasmid was transfected (3 *μ*g) into HeLa cells and lysates immunoprecipitated with Myc antibody or IgG control and immunoblotted for Myc (left panel) and IL-22R (right panel). (b) Immunopurified FBXW12-Myc was probed for association with the SCF constituent Skp-1. (c)* In vitro* ubiquitination assay: IL-22R by itself or with needed cofactors of the ubiquitination reaction (E1 + E2 enzymes and/or Skp-1 and cullin-1) was incubated for 1 h before IL-22R immunoblotting; the presence of the upper band in lane 2 indicates IL-22R ubiquitination. Data are representative of two experiments performed on different days.

To evaluate if FBXW12's effects on IL-22R abundance were due to ubiquitination biochemistry, we investigated interaction between the two molecules. In Figure 2(a), immunoprecipitation of Myc labeled FBXW12 was followed by immunoblot for Myc in nonreducing conditions showing FBXW12 as well as multiple higher molecular weight complexes (left panel) and IL-22R (right panel), indicating that FBXW12 and IL-22R interact in cells. Immunoprecipitated FBXW12-Myc was next interrogated for association with the Skp-1 component of the SCF E3 ligase machinery and reveals* in vitro* association between these molecules as well (Figure 2(b)). We next evaluated direct ubiquitination of IL-22R by FBXW12 in a cell-free system including IL-22R and essential components of the ubiquitination machinery with and without FBXW12 and immunoblot for IL-22R.  Figure 2(c) shows elicitation of the higher molecular weight IL-22R band only when all components of the Ub machinery and FBXW12 are present. Collectively, these data indicate that FBXW12 is a* bona fide* SCF E3 ligase constituent that can bind to IL-22R and other components of SCF to mediate IL-22R ubiquitination.

### 3.3. FBXW12 Knockdown Increases IL-22R and Its Signaling

**Figure 3:**
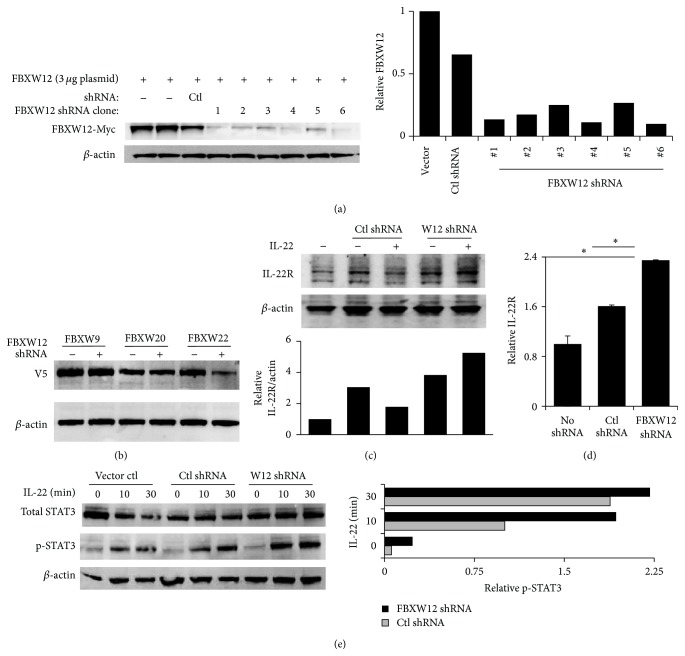
FBXW12 depletion with shRNA increases IL-22R protein levels and activity. (a) HeLa cells transfected with FBXW12-Myc (2 *μ*g) were transiently transfected with shRNA clones 1–6 (Origene) designed to target FBXW12 (2 *μ*g), with reduction in immunoreactive FBXW12. (b) Control or FBXW12 shRNA (clone #4, 2 *μ*g) was cotransfected into HeLa cells with FBXW9, W20, and W22 V5-tagged plasmids with immunoblot for V5. (c) Endogenous IL-22R immunoblot of cell lysates from HeLa cells transfected with control or FBXW12-targeting shRNA (clone #4) in the setting of PBS or IL-22 treatment (60 ng/mL for 60 min). In (d), IL-22R densitometry is shown from 3 shRNA experiments (^*∗*^ *p* < 0.05 by ANOVA for FBXW12 shRNA compared to each other group). (e) Cells transfected with indicated plasmids were treated with IL-22 (60 ng/mL) for the indicated times and lysates probed for activated (Tyr705 phosphorylated) STAT3 with densitometry graphed on the right.

We used a gene silencing approach to further investigate the role of FBXW12 regulation of IL-22 signaling. Plasmids encoding shRNA for FBXW12 and a GFP reporter were transfected into HeLa cells and relative FBXW12-V5 signal is evaluated by immunoblot (Figure 3(a)) with robust depletion of FBXW12 signal seen with all shRNA clones tested. Based on efficacy, shRNA clone 4 was selected for subsequent experiments as it does not significantly decrease ectopically expressed protein FBXW9 or FBXW20 but does deplete the FBXW12 homolog FBXW22 (Figure 3(b)). We observed that HeLa cells express IL-22R protein at low levels, as others have seen for mRNA [19]. Depletion of FBXW12 by shRNA caused an increase in immunoreactive IL-22R under normal resting conditions (Figures 3(c) and 3(d)). Stimulation of cells with IL-22 depletes IL-22R abundance in the presence of control shRNA, but the IL-22R signal is stable in the presence of IL-22 when FBXW12 is depleted by shRNA (Figure 3(c)). Silencing of FBXW12 also causes an enhancement of IL-22 triggered phosphorylation of the signal transducer and activator of transcription 3 (STAT3), a proximal step in IL-22 signaling in epithelia [5] (Figure 3(e)). Together, these data imply that, in cellular physiology, FBXW12 may direct degradation of IL-22R in resting and IL-22 stimulated conditions and that, in the absence of FBXW12, IL-22R abundance and signaling are augmented in epithelial cells.

### 3.4. FBXW12 Functions as a Growth Suppressor

**Figure 4:**
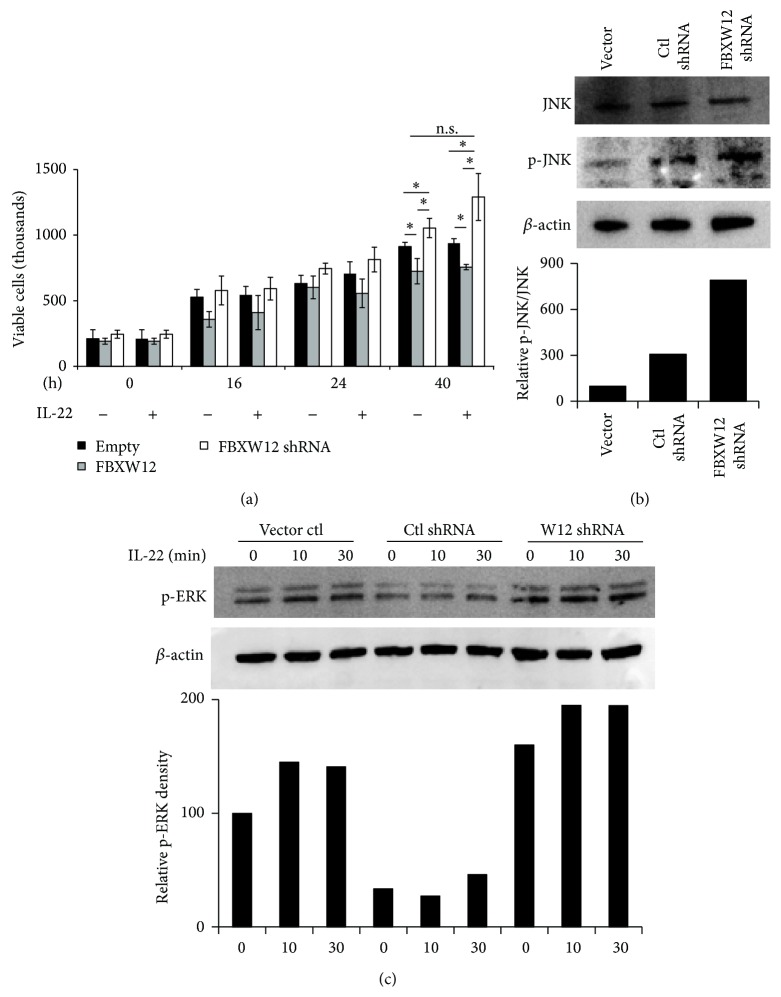
FBXW12 depletion enhances cell proliferation and constitutively increases MAP kinase phosphorylation. (a) HeLa cells were transfected with FBXW12 or shRNA targeting FBXW12 (clone #4 above) and incubated with or without IL-22 (30 ng/mL). Cell counts were performed at indicated times after transfection, and viability was assessed with trypan blue staining (^*∗*^ *p* < 0.05 by ANOVA between all groups within each IL-22 condition at 24 and 40 h for an experiment run with 5 replicates per group). (b) HeLa cells were transfected as above and lysates probed for constitutive phosphorylation of the MAP kinase JNK. (c) Transfected HeLa cells were treated with 60 ng/mL IL-22 for the indicated times and IB was performed for ERK phosphorylation at Thr202/Tyr204.

Because IL-22 signaling is proliferative in epithelial cells and FBXW12 activity blunts this signaling, we wanted to investigate if FBXW12 modulation had any effect on proliferation of epithelial cells. Compared to empty vector, FBXW12 transfection into HeLa cells slowed the growth of cells, whereas transfection of FBXW12 shRNA-containing plasmid caused an increase in cell proliferation over 40 hours as measured by viable cell count (Figure 4(a)). These differences in proliferation were observed in the presence or absence of IL-22 at the 40 h time point (Figure 4(a)). To investigate the mechanism of this proliferative cellular phenotype, we interrogated IL-22-associated second-messenger MAP kinases associated with growth [5, 20]. The proliferation-associated MAP kinases JNK and ERK are more constitutively and inducibly activated in the presence of shRNA targeting FBXW12 (Figures 4(b) and 4(c)).

### 3.5. Suppression of FBXW12 Drives Cell Cycle Progression

**Figure 5:**
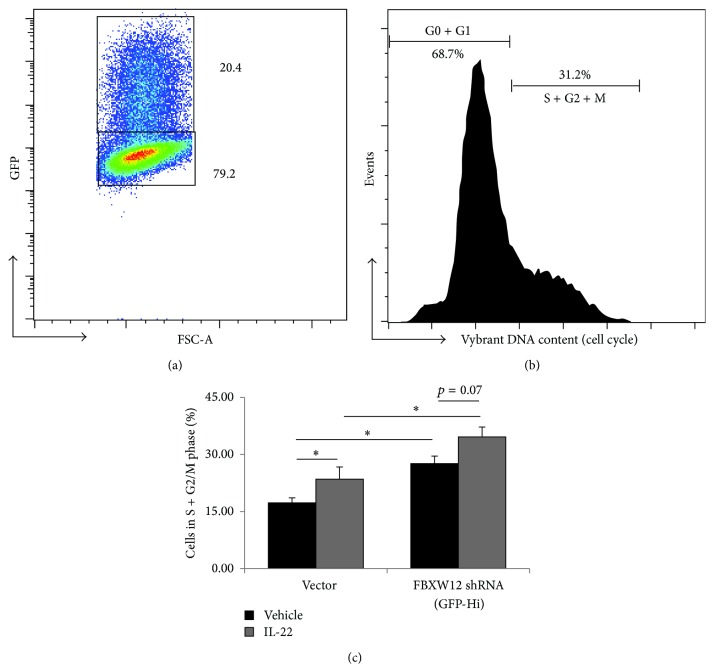
FBXW12 depletion increases cell cycle progression. HeLa cells were transfected with empty vector or FBXW12 shRNA containing a GFP reporter and treated for 8 h with IL-22 or vehicle before DNA staining with Vybrant Dyecycle Ruby for cell cycle analysis. (a) GFP-Hi cells containing FBXW12 shRNA were gated and compared with nontransfected cells. (b) Histograms of cell DNA content were used to analyze cell cycle phase. (c) Proportion of cells with progression out of phase G1 for the indicated groups is shown (^*∗*^ *p* < 0.05 by ANOVA on a single experiment with 4 replicates; data are representative of 4 experiments performed on different days).

We used flow cytometry to analyze cell cycle progression [21] in FBXW12 shRNA-transfected cells compared to nontransfected cells or cells transfected with empty vector. Among cells exposed to empty vector and cells not carrying the GFP transfection marker for FBXW12 shRNA approximately 80% were in the G0/G1 phase with the remainder in the S (≈14%) and G2/M (≈4%) phases. Cells containing FBXW12 shRNA were analyzed based on the presence of the GFP reporter (Figure 5(a), GFP-Hi gating) and display a shift in DNA content signal (Figure 5(b)) with decreased G0/G1 phase cells and increased progression into S/G2/M phases (Figure 5(c)). IL-22 treatment caused an increase in cell cycle progression among the nontransfected cells and a trend in FBXW12 knockdown cells with similar magnitude progression from the G0/G1 population (≈7%). These data corroborate the above findings that FBXW12 functions as a cell growth suppressor and suggest that FBXW12 suppression drives cell cycle progression and cytoproliferation.

## 4. Discussion

In this report, we have identified specific E3 ligase subunits that shuttle IL-22R for degradation in human cell systems. This study is focused on the human protein FBXW12, an F-box family E3 ligase subunit protein that has never been functionally described in any biological system. We demonstrate that FBXW12 causes depletion of overexpressed and endogenous IL-22R. FBXW12 is physically associated both with IL-22R and with components of the SCF E3 ligase apparatus and causes ubiquitination of IL-22R in a cell-free system. We show that knockdown of FBXW12 causes an increase in IL-22R abundance, indicating that FBXW12 is active constitutively for IL-22R processing. Furthermore, shRNA knockdown of FBXW12 enhances IL-22 responsive second-messenger activation with enhanced phosphorylation of STAT3 and MAP kinases. FBXW12 knockdown enhances growth of epithelial cells in both IL-22 responsive and IL-22 independent means, and shRNA to FBXW12 promotes cell cycle progression.

We previously characterized the processing of murine IL-22R through the Ub proteasome system and identified target of residues for ubiquitination of its C-terminus and described IL-22R stabilization by phosphorylation via the glycogen synthase kinase- (GSK-) 3*β* [9]. For human IL-22R we find that FBXW1, FBXW12, FBXW20, and FBXW22 cause a decrease in detectable IL-22R. Other reports have shown that IL-22R family member proteins including the interferon alpha receptor [22] and the IL-10 receptor [23] are targeted for ubiquitination by the well described FBXW1. We did not exhaustively explore if interactions exist between FBXW1 and IL-22R, though this area of investigation may yield interesting results. Human IL-22R is less likely to be phosphorylated by the GSK-3*β* kinase as it has a glycine in place of the aspartic acid residue that constitutes the classical GSK-3*β* consensus sequence [24]. We therefore did not explore if other cross talk exists between FBXW12 and GSK-3*β* relevant to IL-22R abundance or signaling. FBXW12 is the closest related human F-box protein to mouse proteins FBXW20 and FBXW22, and none of these proteins have, to our knowledge, been functionally described as E3 ligase subunits nor have targets been identified for these FBXW proteins. These murine FBXW20 and FBXW22 proteins cause depletion of the murine as well as human IL-22R (data not shown), indicating that the consensus structures for E3 ligase docking and ubiquitination may be shared between species. Since IL-22R persists on the membrane after ligation when FBXW12 is suppressed, FBXW12-dependent ubiquitination of IL-22R may be constitutive or induced after ligation by IL-22 cytokine for receptor internalization and/or degradation.

The FBXW family proteins include many key regulators of inflammation and cell survival. Best described among these are the FBXW1 (*β*-trcp1) and FBXW11 (*β*-trcp2), which have context-dependent roles in cancer and cellular proliferation via the targeting of multiple important transcription factors, second messengers, and receptors which collectively drive proliferative and/or apoptotic phenotypes depending on the cell type and environment [14, 25]. FBXW7 is considered a tumor suppressor that targets cytoproliferative proteins like cyclin E, c-Myc, and Aurora kinases [26]. Our description of a growth suppressive role for FBXW12 in epithelia is therefore consistent with the behavior of these other FBXW family proteins.

The IL-22 signaling axis is essential for host protection at mucosal epithelial surfaces and generates protective responses including antimicrobial peptide release and epithelial growth [3, 4]. Augmentation of this protective IL-22 signaling by FBXW12 is an attractive theme for therapeutic targeting. However, there is a theoretical hazard in FBXW12 antagonism, as unchecked cytoproliferation may drive neoplasia and some authors implicate IL-22 signaling in inflammatory disease [27]. Further development of the pathobiology associated with IL-22R degradation will be an interesting and revealing area for future studies.

## 5. Conclusion

The epithelial proliferative factor IL-22 is an essential component of host defense at mucosal surfaces which acts to fortify the barrier integrity against bacterial invasion. We present a new description of IL-22R processing, wherein IL-22R is shuttled for degradation by the functionally undescribed human protein FBXW12. Increasing FBXW12 suppressed IL-22R levels and IL-22-dependent cellular responses and conversely suppression of FBXW12 increases IL-22R abundance and signaling. Furthermore, FBXW12 seems to act as an endogenous growth suppressor, as its overexpression reduced cell proliferation and FBXW12 knockdown increased epithelial cell growth and drives cell cycle progression. Alteration of IL-22R processing by FBXW12 during acute infection may be therapeutic during infection at mucosal surfaces.

## References

[1] Schulz S. M., Köhler G., Schütze N. (2008). Protective immunity to systemic infection with attenuated *Salmonella enterica* serovar enteritidis in the absence of IL-12 is associated with IL-23-dependent IL-22, but not IL-17. *Journal of Immunology*.

[2] Aujla S. J., Chan Y. R., Zheng M. (2008). IL-22 mediates mucosal host defense against Gram-negative bacterial pneumonia. *Nature Medicine*.

[3] Sonnenberg G. F., Fouser L. A., Artis D. (2011). Border patrol: regulation of immunity, inflammation and tissue homeostasis at barrier surfaces by IL-22. *Nature Immunology*.

[4] Aujla S. J., Kolls J. K. (2009). IL-22: a critical mediator in mucosal host defense. *Journal of Molecular Medicine*.

[5] Lejeune D., Dumoutier L., Constantinescu S., Kruijer W., Schuringa J. J., Renauld J.-C. (2002). Interleukin-22 (IL-22) activates the JAK/STAT, ERK, JNK, and p38 MAP kinase pathways in a rat hepatoma cell line. Pathways that are shared with and distinct from IL-10. *Journal of Biological Chemistry*.

[6] Xie M.-H., Aggarwal S., Ho W.-H. (2000). Interleukin (IL)-22, a novel human cytokine that signals through the interferon receptor-related proteins CRF2-4 and IL-22R. *The Journal of Biological Chemistry*.

[7] Wolk K., Kunz S., Witte E., Friedrich M., Asadullah K., Sabat R. (2004). IL-22 increases the innate immunity of tissues. *Immunity*.

[8] Pociask D. A., Scheller E. V., Mandalapu S. (2013). IL-22 is essential for lung epithelial repair following influenza infection. *The American Journal of Pathology*.

[9] Weathington N. M., Snavely C. A., Chen B. B., Zhao J., Zhao Y., Mallampalli R. K. (2014). Glycogen synthase kinase-3*β* stabilizes the interleukin (IL)-22 receptor from proteasomal degradation in murine lung epithelia. *Journal of Biological Chemistry*.

[10] Glickman M. H., Ciechanover A. (2002). The ubiquitin-proteasome proteolytic pathway: destruction for the sake of construction. *Physiological Reviews*.

[11] Ravid T., Hochstrasser M. (2008). Diversity of degradation signals in the ubiquitin-proteasome system. *Nature Reviews Molecular Cell Biology*.

[12] Jin J., Cardozo T., Lovering R. C., Elledge S. J., Pagano M., Harper J. W. (2004). Systematic analysis and nomenclature of mammalian F-box proteins. *Genes & Development*.

[13] Frescas D., Pagano M. (2008). Deregulated proteolysis by the F-box proteins SKP2 and *β*-TrCP: tipping the scales of cancer. *Nature Reviews Cancer*.

[14] Fuchs S. Y., Spiegelman V. S., Kumar K. G. S. (2004). The many faces of *β*-TrCP E3 ubiquitin ligases: reflections in the magic mirror of cancer. *Oncogene*.

[15] Tan Y., Sangfelt O., Spruck C. (2008). The Fbxw7/hCdc4 tumor suppressor in human cancer. *Cancer Letters*.

[16] Mallampalli R. K., Coon T. A., Glasser J. R. (2013). Targeting F box protein Fbxo3 to control cytokine-driven inflammation. *Journal of Immunology*.

[17] Weathington N. M., Mallampalli R. K. (2014). Emerging therapies targeting the ubiquitin proteasome system in cancer. *Journal of Clinical Investigation*.

[18] Chen B. B., Mallampalli R. K. (2009). Masking of a nuclear signal motif by monoubiquitination leads to mislocalization and degradation of the regulatory enzyme cytidylyltransferase. *Molecular and Cellular Biology*.

[19] Sauane M., Gopalkrishnan R. V., Lebedeva I. (2003). Mda-7/IL-24 induces apoptosis of diverse cancer cell lines through JAK/STAT-independent pathways. *Journal of Cellular Physiology*.

[20] Chang L., Karin M. (2001). Mammalian MAP kinase signalling cascades. *Nature*.

[21] Nunez R. (2001). DNA measurement and cell cycle analysis by flow cytometry. *Current Issues in Molecular Biology*.

[22] Kumar K. G. S., Krolewski J. J., Fuchs S. Y. (2004). Phosphorylation and specific ubiquitin acceptor sites are required for ubiquitination and degradation of the IFNAR1 subunit of type I interferon receptor. *Journal of Biological Chemistry*.

[23] Jiang H., Lu Y., Yuan L., Liu J. (2011). Regulation of interleukin-10 receptor ubiquitination and stability by beta-TrCP-containing ubiquitin E3 ligase. *PLoS ONE*.

[24] Sutherland C. (2011). What are the *bona fide* GSK3 substrates?. *International Journal of Alzheimer's Disease*.

[25] Lau A. W., Liu Y., Tron A. E., Inuzuka H., Wei W. (2014). The role of FBXW subfamily of F-box proteins in tumorigenesis. *SCF and APC E3 Ubiquitin Ligases in Tumorigenesis*.

[26] Welcker M., Clurman B. E. (2008). FBW7 ubiquitin ligase: a tumour suppressor at the crossroads of cell division, growth and differentiation. *Nature Reviews Cancer*.

[27] Brand S., Beigel F., Olszak T. (2006). IL-22 is increased in active Crohn's disease and promotes proinflammatory gene expression and intestinal epithelial cell migration. *The American Journal of Physiology–Gastrointestinal and Liver Physiology*.

